# Developing shared understandings of recovery and care: a qualitative study of women with eating disorders who resist therapeutic care

**DOI:** 10.1186/s40337-016-0114-2

**Published:** 2016-12-16

**Authors:** Connie Musolino, Megan Warin, Tracey Wade, Peter Gilchrist

**Affiliations:** 1 Gender Studies & Social Analysis, School of Social Sciences, Faculty of Arts, University of Adelaide, Napier Building, Adelaide, South Australia 5005 Australia; 2 School of Psychology, Flinders University, Adelaide, South Australia Australia; 3 Psychiatrist in private practice, Adelaide, South Australia Australia

**Keywords:** Eating disorders, Recovery, Care, Therapeutic relationship, Cultural context, Qualitative

## Abstract

**Background:**

This paper explores the differing perspectives of recovery and care of people with disordered eating. We consider the views of those who have not sought help for their disordered eating, or who have been given a diagnosis but have not engaged with health care services. Our aim is to demonstrate the importance of the cultural context of care and how this might shape people’s perspectives of recovery and openness to receiving professional care.

**Method:**

This study utilised a mixed methods approach of ethnographic fieldwork and psychological evaluation with 28 women from Adelaide, South Australia. Semi-structured interviews, observations, field notes and the Eating Disorder Examination were the primary forms of data collection. Data was analysed using thematic analysis.

**Results & Discussion:**

Participants in our study described how their disordered eating afforded them safety and were consistent with cultural values concerning healthy eating and gendered bodies. Disordered eating was viewed as a form of self-care, in which people protect and ‘take care’ of themselves. These subjectively experienced understandings of care underlie eating disorder behaviours and provide an obstacle in seeking any form of treatment that might lead to recovery.

**Conclusion:**

A shared understanding between patients and health professionals about the function of the eating disorder may avoid conflict and provide a pathway to treatment. These results suggest the construction of care by patients should not be taken for granted in therapeutic guidelines. A discussion considering how disordered eating practices are embedded in a matrix of care, health, eating and body practices may enhance the therapeutic relationship.

## Background

It is well recognised that recovery is a contested term in the eating disorder literature and that ‘there is no single definition or description of [this concept]’ ([1] p4). A number of studies point to inconsistencies with the way criteria for recovery is used and defined in clinical trials, making it difficult to compare research and reach consensus [2, 3]. Current clinical definitions of recovery incorporate the presence of minimal eating disorder psychopathology (i.e., within one standard deviation of the range of healthy populations), the absence of disordered eating behaviours, and achievement of a healthy body mass index [3].

There has been a movement towards recovery-oriented practice and service delivery [4]. Those with lived experience of a mental illness and support organisations have emphasised the recovery model primarily within a social justice movement aimed at restoring the human rights and full community inclusion of people with mental health issues [1]. Australia’s *National Framework for Recovery-Oriented Mental Health Services* reflects this momentum, recognising the value of lived experience, the diffuse lines of recovery, and respecting clients’ knowledge and choice alongside that of health professionals [1].

The recovery model is a central theme in the *Royal Australian and New Zealand College of Psychiatrists Clinical Practice Guidelines for the Treatment of Eating Disorders* [5]. It is intended to provide current evidence based guidance on the assessment and clinical treatment of people with eating disorders in the Australian and New Zealand context [5]. The guidelines state ‘care for people with eating disorders should be provided within a framework that supports the values of recovery-oriented care’ ([5] p983). This document for the clinical management of eating disorders has been well received and represents the work of a collaboration of health care academics and professionals, and wide consultation with key stakeholders and the community. In their systematic review Hay et al., point out that ‘most people make a sustained recovery with treatment’, including ‘people with anorexia nervosa, where up to 40 % of adults (and a higher percentage of adolescents) will make a good five-year recovery, a further 40 % a partial recovery and those with persistent illness may yet benefit from supportive therapies’ ([5] p979). Research indicates that 50 % of those with bulimia nervosa fully recover and the outcomes with treatment for binge eating disorder obtain similar results [5].

However, Ben-Tovim et al.’s highly cited study on eating disorder outcomes in South Australia [6] concludes that ‘many patients make a good recovery without accessing specialised treatments of any kind’, including treatments such as lengthy admissions for weight gain or long-term outpatient care, pointing to the need to explore other contributing factors in people’s lives ([6] p1257). The course of natural recovery may differ depending on the eating disorder, with one study finding that 5-year prognosis for bulimia nervosa was poor, while the majority of those people with binge eating disorder were recovered [7]. Other studies have found that it is common for presentation for treatment to occur many years after onset of an eating disorder, and into late middle-age [5, 8, 9], highlighting that a large population of people with eating disorders are not engaged with treatment. These findings point to the diversity of recovery experiences, and to the importance of exploring qualitative experiences of disordered eating and recovery to understand what impedes and encourages recovery.

There are a growing number of qualitative studies on recovery from eating disorders [10–15] that focus on patient perspectives. Such studies also identify obstacles to recovery. For example, qualitative studies show that the pursuit of low weight addresses a sense of ineffectiveness, makes the person feel safe, helps communicate distress related to possible rejection and abandonment, and moderates the experience of negative emotions [11, 13, 16]. Bjork and Ahlstrom argue that qualitative approaches allow for different dimensions to be explored that would risk being lost in quantitative research. In their qualitative study of patient’s experiences of recovery from chronic anorexia nervosa, Dawson et al. note that the accounts of the women they interviewed should be understood within their gendered and cultural context [14]. While the women ‘did not greatly examine the sociocultural context from which their AN developed and recovery took place’ ([14] p503), Dawson et al. suggest that such investigations would deepen understandings of the cultural processes that underpin eating disorders. Similarly, in her analysis of gender and recovery in eating disorders, Moulding argues that while qualitative studies draw attention to the cultural dimensions of recovery, ‘there is [actually] little attention to the social dimensions of these processes, with the focus primarily on intrapsychic factors’ ([15] p71) located within individuals.

The national framework on recovery acknowledges the subjective experiences of recovery beyond medical and psychiatric classification, with a focus on collaboration between people with disordered eating, carers and health professionals. However, the recovery model does not currently engage with people's cultural perceptions and experiences of eating and care, despite the aim of recovery-oriented treatment being to encourage people to seek professional health care and practice self-care. The national framework includes sections on ‘understanding cultural idioms’ and ‘keeping diversity in mind’ which focus on people from culturally and linguistically diverse backgrounds; Aboriginal and Torres Strait Islanders; refugees and asylum seekers; LGBTI people; and other minority groups [1]. In the eating disorder therapeutic guidelines, an exploration of culture is limited to the inclusion of the section *Indigenous care*, *a dimensional and culturally informed approach to diagnosis and treatment* ([5] p983). Culture is not an external attribute or independent variable (such as one’s ethnicity), but involves the myriad of taken-for-granted and embodied practices that give meaning to our everyday worlds. Anthropologists have long pointed out that culture is practiced through ‘the shared … (implicit and explicit) values, ideas, concepts, and rules of behaviour that allow a social group to function and perpetuate itself’ ([17] p345). All groups and societies (including researchers and health care professionals) have a number of co-existing, overlapping and competing subcultures [17]. Leading cultural psychiatrists (e.g., Kirmayer and Minas 2002) and the recent Lancet Commission on culture and health [18] support the view that culture is fundamental both to the causes and course of psychopathology and also to the effectiveness of systems of healing and health care. Population health literature also suggests that social factors, rather than medical interventions, are the main determinants of recovery from mental ill-health [19–21] (see also [22] for concept of ‘recovery capital’).

Therefore, while the recovery-oriented framework for treatment promotes inclusive service delivery, it lacks an interrogation of the cultural contexts of recovery and care. The main aim of this paper is to explore the cultural contexts in which a person experiences an eating disorder and how this is critical to how they approach recovery. Healthy eating and lifestyle discourses act as ubiquitous cultural signposts for people wishing to maintain eating disorder practices (‘watch what you eat’, ‘you are what you eat’) and often compete with medical and psychiatric advice. Dutch anthropologist Annemarie Mol has written widely about eating, bodies and care practices in health care settings [23]. Her ‘logic of care’ is a useful framework to discuss how understandings of care and recovery might differ between people with eating disorders and practitioners – and why people might not seek therapeutic care in the initial phases of disordered eating or in the case of severe and enduring eating disorders [24]. In the RANZCP 'Clinical Practice Guidelines for the Treatment of Eating Disorders', ‘meaningful engagement in therapy’ is singled out as being ‘a crucial component in all treatments for anorexia nervosa’ ([5] p988). Expanding on what ‘meaningful engagement’ looks like in practice would be beneficial, and we argue a framework of care may be valuable for thinking through the different understandings of care held by patients and practitioners. Furthermore, the national framework on recovery offers insights which could be expanded to include a discussion on perspectives of care. These include the framework urging health professionals to be aware of ‘a person’s explanatory models of illness, distress and wellness’ and ‘the impact of the practitioner’s own language, cultural beliefs and values on the therapeutic relationship barriers to service’ ([1] p14). A therapist’s capacity to understand how a person with disordered eating may perceive their practices as a form of self-care and health [25, 26] is an example of recognising an individual’s explanatory model and personal agency.

A recently commissioned report found that of the one million Australians who suffer from an eating disorder, less than 30 % engage with treatment [27]. Research to date has mainly focused on people who engage with treatment services [28], but we know very little about the significant number of people who do not seek help, or delay seeking help for many years. This paper thus offers new insights into why people might not even consider accessing recovery pathways, or take many years to do so. The results reported in this study are part of a larger project that aimed to identify why people with eating disorders deny they have a problem, or delay and resist professional care. In working with a group who are significantly under-researched, we aimed to demonstrate how behaviours were rationalised as part of a cultural milieu in which care of one’s self, demonstrated through careful eating and physical exercise, was culturally legitimated and widely sanctioned. In attending to how people understand their behaviours (as normal and ‘not sick’), we hypothesized that this would illuminate important cultural contexts that underpin and potentially obfuscate a need to attend to recovery.

## Methods

### Participants and recruitment

Data collection occurred over 15 months (January 2013 to March 2014) in Adelaide, South Australia and involved 28 women, ranging in age from 19 to 52. The criteria for recruitment included women who were over 16 years of age and had not seen a health professional for disordered eating, had not been given an eating disorder diagnosis, or had been diagnosed but had delayed seeking treatment or did not wish to pursue treatment.

Participants were recruited through snowball sampling methods, with posters being placed around two metropolitan university campuses. The majority of posters were placed on the backs of toilet doors and posed questions such as ‘Are you continually thinking about your food and your weight?’ and ‘Do you enjoy the feeling of not eating or excessive exercising?’. Privacy was crucial to the locations of the recruitment information due to the social stigma associated with eating disorders and the nature of this study seeking participants who have not previously disclosed their eating issues. This allowed the potential participant to seek out information on the study privately by emailing or phoning Author 1. As this was a difficult sample to recruit, participants were also recruited through mental health networks and advertising on social media websites such as Facebook groups South Australian Body Esteem Activists and Supporting Eating Disorders for South Australia. Most of the recruited women were under 30 years of age, university students and of Anglo-Australian backgrounds.

### Design

Through a mixed methods approach including ethnographic fieldwork and psychological evaluation, this study focused on examining the cultural contexts of women, food and disordered eating, with the aim of developing strategies for early intervention. The research team was multidisciplinary, and included a social scientist, a medical anthropologist skilled in gender analysis, and a psychiatrist and psychologist (both of whom specialised in eating disorders). In taking a multidisciplinary approach, the authors attempted to re-examine the experience of eating disorders not from a clinical or tertiary point of view, but from a mixed method approach framed by a sociocultural perspective. This approach led to a questioning of taken-for-granted concepts such as health, illness, eating and recovery, not only providing a platform for exploring how these categories are culturally constituted, but also providing a framework for questioning the categories that underpin therapeutic understandings of recovery and care.

### Data collection

Data collection began with a pilot phase that included three women who partook in at least 2 semi-structured interviews, the Eating Disorder Examination (EDE) and a diary writing phase. The pilot interviews gave Author 1 and 2 the chance to collaboratively reflect on the interview schedule and seek participants’ feedback, adapting the study design where possible.

From the pilot phase the research team deduced that the most appropriate order for conducting the interviews was to begin with a semi-structured interview in the first meeting (allowing for rapport to be built with the participant). In the second meeting the EDE was administered in order to ascertain if participants might fit the diagnostic criteria of an eating disorder. The inclusion of the EDE was important to examine how participants responded to such evaluations, and provide them with information for resources and services. EDE results were sent to a researcher trained in the use of the EDE (who analysed the data using SPSS and reported back to the team). The third meeting began with a debriefing session about the EDE, and then continued with the semi-structured interview. The interviews were guided by an interview schedule, which asked questions that explored what type of practices participants engaged in on a daily basis (i.e. how they ate, exercised, engaged in activities); if they considered their activities ‘a problem’; what cultural ‘norms’ helped to support their eating and exercise activities; and if they had ever considered seeking help. Due to the exploratory nature of qualitative research, the interview schedule was flexible and follow-up interviews with each participant provided opportunities to explore their everyday lives in more detail. In total, sixty-eight semi-structured interviews took place in people's homes, in interview rooms at one of the universities, in cafes and in public places.^1^ In addition, recruitment for this study could be slow and some participants were non-responsive. Four of the women who partook in one or two interviews stopped responding to Author 1’s efforts to schedule more interviews. In attempting to locate a population that does not identify as having ‘a problem’, faces social stigma, and is reluctant to come forward and engage with services, the recruitment and data collection processes highlight issues of accessibility and privacy with such a hard to reach group.

Semi-structured interviews and observation are key methods of data collection in ethnographic and qualitative approaches to research. Field notes taken during and after interviews were critical to data collection as they captured observations made during the research encounters, such as non-verbal cues, emotional reactions performed through bodily dispositions, appearances, the research setting, as well as reflexive notes on how the researcher may react to the participant’s narrative (which adds to research rigor including research bias and how the researcher may impact the research process) [29].

As disordered eating is associated with secrecy and shame, participants were also given the opportunity to engage in a diary writing phase for 8 weeks, in which they wrote about the everyday moments, activities or events that supported their disordered eating behaviours, and their fears, pleasures and desires around food and their body. Collecting diaries from participants also gave Author 1 another opportunity to discuss the research experience with the participant.

### Analysis

Grounded theory principles guided the research methods, coupled with thematic techniques of data collection and analysis [30, 31]. Grounded theory is a qualitative approach which prioritises deriving analytic categories and themes directly from the data, not from pre-conceived concepts or hypotheses [30]. All interviews (including semi-structured and EDE interviews) were professionally transcribed, and field notes were written up following each interview. To become closer to the data Author 1 transcribed the pilot interviews and open coded them within the same week afterwards. During the pilot phase of the study a list of codes were developed around certain themes, for example, ‘help seeking’, ‘food’, ‘protection’, ‘ambivalence’, to then form the basis of the thematic analysis of the interview and diary data. Following the established coding process of open, axial and selective coding, the interview manuscripts and field note data was firstly open coded on the computer in a Word document, and then through the software programme NVivo by Author 1. Open coding involved reading the transcripts and diaries line by line to identify and develop any ideas, themes or issues from the data [29]. In the collaborative meetings that followed between Author 1 and 2, axial (or secondary) codes were developed. This stage of data analysis involved making comparisons across the data, so that the final stage of selective coding could occur. Selective coding involved taking core themes and positioning these as key theoretical frameworks for analysis, and critically examining their concordance (or not) with the wider literature.

## Results

### Participant descriptive

**Fig. 1:**
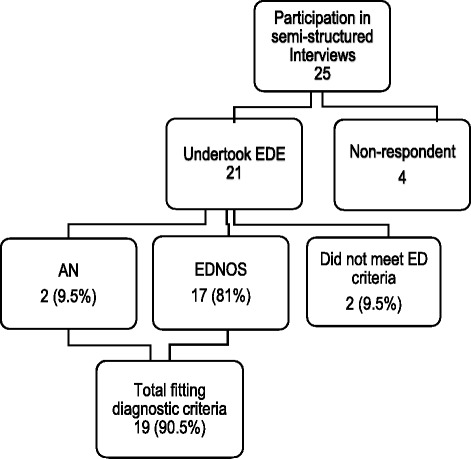
Participant diagnosis

Of the 21 participants who consented to undertake the EDE (N = 21), the mean global EDE score was 3.48 (SD = 1.06), with a range from 0.92 to 5.57. The majority (90 %) met criteria for an eating disorder. Most (81 %) fell into the Eating Disorders Not Otherwise Specified (EDNOS) category, and 2 met the diagnostic criteria of anorexia nervosa (See Fig. 1). Of the total sample who participated in the semi-structured interviews (25), six had a previous eating disorder diagnosis (anorexia nervosa) from a health care professional, and had had varying, but limited contact with health providers, and no desire to recover (in clinical terms). The other nineteen participants had not previously sought professional help and had never received a diagnosis.

As shown in Table 1, participants self-reported when they believed their disordered eating begun and for most participants, issues had begun in childhood and adolescence. While experiences differed greatly, we report on two key findings (disordered eating as producing safety, and culturally dominant ideals of health) that are both understood as practices of care, thereby negating the need for therapeutic care.

**Table 1:** Self-reported length of disordered eating

Not reported	Under 1 year	2 + years	5 + years	10 + years	20 + years
5	1	2	7	6	4
18 %	3.5 %	7 %	25 %	21.5 %	14 %

### Disordered eating is perceived as ‘safe’

People’s experiences of disordered eating were often described as ‘safe’. Maintaining safe spaces, doing safe things (like having the same plate to eat from day after day), maintaining routines and eating ‘safe foods’ were common themes. Forty five year old Morgan (who has experienced 30 years of eating disorders) said: ‘It’s safest *not* to have too much variety: more variety seems to make you hungrier or something. It’s weird’. Another participant aged in her 50s who had lived with eating disorders for 30 years (and had enduring anorexia) described the safety and comfort that her practices afforded her:

*the ritualistic side of it where you feel safe if you’re sticking to your normal, you know, that’s why you do it … you feel safe if you know what to expect if you stay on this sort of a routine and a diet.*

Twenty year old Lucy, who had developed disordered eating at age 12 and never sought help (and whose EDE revealed EDNOS), similarly described her experiences as ‘kind of safe’ – yet recognised the contradictory nature of safety and suffering that she endures:

*There is kind of two sides to it I guess, it’s like comforting but it’s also exhausting at the same time.*

*Yeah. So what’s kind of comforting about it?*

*I guess just, I guess because if I don’t follow what I do, I feel like really panicky and anxious and like a bit depressed and stuff. But if I sort of stick to it, it just makes me feel sort of more calm even if I am sort of tired and everything.*

Michelle (aged 27), who had swung between a diagnosis of anorexia and EDNOS for more than 10 years stated that the only time she feels ‘okay’ about herself, is when she is ‘sticking to [her] routines’. Her routines involve only eating safe foods (‘lettuce and stuff like that’) in order to create safety:

*it is very, very much a safe space and almost like a, I guess being invincible almost, like nothing can touch me while I’m here, like I’m managing to do this and I’m managing to stick through it all. So yeah, “What can really defeat me if I’m living on nothing?” if that makes any sense at all…*

This strong sense of safety (which was sometimes described as comfort, control or familiarity) was contrasted with the fear of seeking treatment. Some said they were ‘petrified’ of seeing a psychiatrist, because ‘only crazy people see psychiatrists’. Others said ‘I don’t think my eating is a problem’ and ‘it’s not an illness … it’s only a food thing’. Charlotte (who had travelled to the US for treatment) explained that going into treatment was anxiety provoking as it was an exercise in ‘fattening up’, where the primary focus was on weight gain as an indicator of wellness.

*I refused to go somewhere where I would be monitored at that level. I was over that, I found it humiliating, I wasn’t going to go there and they do the whole you know fatten you up, kick you out type thing.*

Because her eating disorder was such a safe and familiar space for over 17 years, Charlotte was unsure if recovery was even possible: ‘I’m conflicted because I know that you can recover to a point, you know after a long journey … but then I also know or discovered that you can be almost ED free for a number of years and think it’s totally behind you, and then something happens and it’s old and familiar’.

Clinicians and therapists will be familiar with this characterisation of eating disorders as ‘safe spaces’. Ethnographic work by Author 2 has also highlighted the ways in which people describe anorexia as a ‘safety net’ ([32] p90), a ‘safe place’ ([32] p128), ‘the thing you believe is keeping you safe’ ([32] p186). Other anthropologists have similarly noted the ways in which people talk about the protective spaces of anorexia, as ‘my little bubble’ ([16] p97); as something that guards me … from the world, from people … something of my own that protects me’ ([33] Eli, forthcoming). Thus, as Lavis suggests, the perceived safety of disordered eating ‘offers a way of caring for the self that navigates tensions … [it] looks after you’ ([26] p98).

### Recovering in a culture where an obsession with thinness and dieting is the norm

The women in our study highlighted how cultural understandings of healthy eating and exercise (the constant bombardment of cultural imagery that thin is healthy and self-discipline is morally superior), made the impetus towards recovery appear somewhat contradictory and defeating. Women remain disproportionately diagnosed with eating disorders, and cultural preferences for thin, weight-managed female bodies are deeply embedded and valued in most western cultures. This bodywork, as Hardin [34] and others note, is highly gendered and informs everyday cultural practices around food and eating. Charlotte explained during an interview: ‘I found at one point when I was doing really well that I was recovered to the point where I had a healthier relationship with food and body than every other normal woman around me. And that was really disturbing. And really challenging’. She constantly struggled with all the information about what foods one should and shouldn’t eat, and the imperative to take care of one’s self through the making the right, healthy choices:

*I kept going back to the pantry, trying to find something that fit the criteria that would be okay to eat. And I could discount everything in the pantry for one reason or another, based on antioxidants, or fibre or glycaemic index, or the level of refinement or preservatives, or colourings or sugars or, you know? There wasn't a single thing in that pantry that was okay, if I put all of our society's messages and health professionals’ advice together about what's okay and what's healthy to eat*.

Rochelle demonstrated the contradictions imbued in being healthy and ‘normal’, revealing that recovery does not occur in a vacuum but rather in a particular gendered and cultural context. She said:

*There's so much health promotion but how much of its healthy, it’s difficult to say. I once read that recovery isn’t like going into a healthy lifestyle and being able to eat foods with fat, having that anxiety and things like that and when you look at Michelle Bridges* 2 *and all those 12 week things, your whole day is still centred around food and I’ve tried to do those kind of things but it’s like I still get the anxiety.*

Several scholars have noted the ways in which people with eating disorders hide their practices within normative cultural ideals around food and bodies [25, 34]. This might be through excuses about food allergies, special diets or intolerances, and the pursuit of health enhancing activities and self-discipline (such as wearing Fitbits) that are culturally valued and understood to demonstrate moral virtue. During an interview Sarah joked how easy it was to continue her excessive exercise routine in a 24-h gym where no one looked sideways at her because ‘most of the people there are like high risk for heart attacks, on steroids and things’. The acceptance of constantly working on and pushing one’s body to extremes was normalised and accepted as part of the visible performance of bodily discipline and virtue.

In a time when fatness is stigmatised and associated with ill health and deviance [35–37], LaMarre and Rice suggest that ‘adding body size to the recovery equation highlights difficulties with following prescriptions for recovery in a society that positions weight gain as wholly negative’ ([38] p138). Participants in Malson et al.’s study pointed to the ‘culturally constituted tension between, on the one hand, treatment goals of reducing weight concerns and, on the other, culturally normative idealisations of slenderness and the near-ubiquity outside of the eating disorder ward of body image concerns’ ([39] p29). Moreover, setting goals towards weight gain or target weights, while obviously vital to survival and cognitive functioning, are seen as antithetical to current cultural discourses about weight reduction as taking care of one’s health. As Tamara explained, ‘I think it can be even more painful when you are weight restored but people don’t understand that you’re still suffering’. These examples demonstrate how it is important to understand recovery in its cultural context, including how disordered eating practices are intimately entangled in gendered practices of care and healthy lifestyles.

## Discussion

To maintain disordered eating, participants engaged in high levels of self-discipline, and found pleasure in the perceived safety that starving, bingeing and purging afforded. Participants felt protected, and in doing so, they took care of themselves by not having to care, not having to feel. Unlike physical illnesses, disordered eating was described as serving a purpose: ‘Like if you break your arm you know something is wrong whereas when you have an eating disorder you’re doing it to escape from something else’. This escape was often a distancing from gendered trauma, of sexual abuse and violence. For Sarah, childhood abuse and neglect led to ‘playing with food’ as a way to ‘distract’ and ‘switch everything off’. Starving, was thus positioned as a way to keep her safe from ‘dangerous’ circumstances in which ‘someone might have an interest in you that is sort of not what you want’.

Understanding people’s experiences of how disordered eating is a form of care is key to why people may not come forward to engage in professional care. A critical exploration of the multiple meanings of care; the daily practices of care giving and experiences of receiving care, may provide insight into the tensions discussed above. For participants in our study good care was often talked about and formed a rationalisation for not seeking therapeutic care [25]. Care was being on a strict raw food vegan diet to prevent obesity. Care was bingeing on junk food as a reward for weeks and months of extreme restricting. Care was only consuming a liquid diet because solid food brought on a desire to binge. Care was starving and shrinking the body to repel unwanted sexual attention. Eating disorders were practiced through careful attention to changing bodies, surroundings, tastes, textures, desires, hunger and relationships. For the therapist, carer, family member and friend, Winace argues that ‘to care is to be sensitive to the attachments that support people, attachments which are sources of both constraints and opportunities, which are openings and closures’ ([40] p110). Being attentive to the way people experience different modalities of care through their disordered eating practices presents possibilities for therapists to broaden their practices of good care and nurture a therapeutic relationship.

If we take Lavis's contention that ‘caring is cyclical as care of self necessarily instigates caring for [the eating disorder] so that it may continue to ‘look after you’ ([26] p104), we can begin to understand ‘the sense of being cared for by the illness’ ([16] p71). In taking this insightful premise, the disordered eating becomes not just a problem of the individual patient, but part and parcel of one’s social world. Thus wider cultural factors are brought to bear, and can be used to broaden current understandings of eating disorders beyond ‘egosyntonic disorders’ ([41] p845).

It is critical for the development of a good therapeutic relationship to broaden our understanding of obstacles to recovery within the recipients of treatment, which can portray the client as “‘hostile’, ‘oppositional’, ‘uncooperative’, and ‘impervious to treatment’” ([39] p26), to an understanding of people’s experiences of self-care and health. Boughtwood and Halse argue ‘tension between patients and clinicians over treatment can undermine the therapeutic relationship, which is the social contract between patient and clinician to communicate and collaborate on their shared goals and objectives for treatment’ ([42] p84). Furthermore, they point out that the literature on the therapeutic relationship is written largely from the perspective and goals of researchers and clinicians with the aim of improving treatment and identifying variables affecting treatment outcomes [42]. The voices and experiences of those living with disordered eating in clinical and research settings therefore may offer valuable insight into why from their perspective the therapeutic relationship and treatment is failing.

Mol’s logic of care proposes that ‘patient choice’ and ‘good care’ often clash in health care environments, and instead of pitting choice and care against each other, Mol views care practices as attending to ‘the unpredictability’s of bodies with disease’, rather than a battle for control ([24] p14). It could be said that it is the daily practices of ‘good care’ that become important to strive for in cases of severe and enduring eating disorders rather than expectations of medical recovery. This is somewhat acknowledged in the proposal for a harm minimisation approach which centres on improving quality of life and reducing distress rather than focusing on symptom reduction [14, 43].

In his keynote presentation to the Australian and New Zealand Academy for Eating Disorders 2015 conference, Ivan Eisler called for ‘a shift from control to caring’. He discussed how within Family Based Therapy there needs to be a focus on getting parents and carers to ‘care better’ instead of focusing on taking control of their children’s eating. This highlights how often in eating disorder institutions good care has come to signify control of patients; control of their bodies, consumption, spaces and routines. Broughtwood and Halse argue such approaches define patients by their eating disorder behaviours and that it would create greater understanding in the therapeutic relationship if instead clinicians attended to individuals’ ‘creative negotiations of hospital practices’ and assisted patients ‘in utilising their creativity to confront their illness in positive ways’ ([42] p92). Therefore, it may be useful to approach the actions of people with disordered eating through a prism of care, rather than an escalation of control measures when patients present as ‘difficult’. This is consistent with recent research from inpatient settings in Montreal which illustrate that autonomous motivation was a significant predictor of change in severity of eating symptoms and attitudes such that patients with higher pre-treatment levels of autonomous motivation showed larger posttreatment reductions on these indices [44]. No such effects were associated with controlled motivation. It is also consistent with seminal work of Touyz and colleagues, which showed that a lenient program for anorexia nervosa did not have poorer results than a strict operant program [45].

If the focus is on controlling the patient, the body or the symptoms, greater emphasis will be placed on the failures of the person or clinician involved. Hay et al. argue ‘because patients with anorexia nervosa are extremely ambivalent about therapy and have starvation related cognitive deficits, current change-oriented treatments may actually be counterproductive and give patients another experience of failure rather than being helpful’ ([46] p1142). Mol conceptualised the logic of care as a way to practice and view care in a way that ‘does not impose guilt, but calls for tenacity’ and ‘for a sticky combination of adaptability and perseverance’ ([24] p91). Such an approach to care giving may be useful for those with severe and enduring experiences of disordered eating.

## Conclusion

This paper has explored how differing perspectives of care hinder shared understandings of recovery. The women in our study highlight how dominant models of recovery take-for-granted and overlook the ways in which the safe spaces of disordered eating and attention to healthy lifestyle mantras are in themselves, a form of care. These differences become a barrier to seeking therapeutic care and recovery. In addition, the women’s narratives demonstrate how recovery is tied to subjective experiences and embedded in one’s cultural environment, not just treatment of medical and psychiatric symptoms. It is important to acknowledge that for people with disordered eating, their practices can be seen through a lens of self-care, in which recovery thus becomes positioned as unnecessary. Our work confirms the findings of Lavis's UK study with women diagnosed with anorexia, in which she found that ‘although self-starvation may be clinically framed as an expression of a lack of self-care, it emerges from informants’ narratives as a modality of self-care that is simultaneously a response and precarious solution to pain’ ([16] p68).

## Footnotes

1 At a later stage in the project we conducted a focus group with a small number of women who considered themselves to be in recovery. The purpose of this focus group was to ask the women about strategies for early intervention. We do not report on the focus group findings in this paper.

2 Michelle Bridges is a personal trainer on the Australian version of ‘The Biggest Loser’ and has various weight loss products (including a 12 week body transformation program).

## References

[1] Commonwealth of Australia. A national framework for recovery oriented health services. Australian Health Ministers Advisory Council. 2013. Available at: www.health.gov.au/internet/main/publishing.nsf/content/67D17065514CF8E8CA257C1D00017A90/$File/recovgde.pdf (accessed 11.12.2015).

[2] Williams SE, Watts TKO, Wade TD (2012). A review of the definitions of outcome used in the treatment of bulimia nervosa. Clin Psychol Rev.

[3] Bardone-Cone AMH, Maldonado CR, Lawson MA (2010). Defining recovery from an eating disorder: conceptualization, validation, and examination of psychosocial functioning and psychiatric comorbidity. Behav Res Ther.

[4] Dawson L, Rhodes P, Touyz S (2014). The recovery model and anorexia nervosa. Aust N Z J Psychiatry.

[5] Hay PJ, Chin D, Forbes D (2014). The royal Australian and New Zealand college of psychiatrists clinical practice guidelines for the treatment of eating disorders. Aust N Z J Psychiatry.

[6] Ben-Tovim DI, Walker K, Gilchrist P (2001). Outcome in patients with eating disorders: a 5-year study. Lancet.

[7] Fairburn CG, Cooper Z, Doll HA (2000). The natural course of bulimia nervosa and binge eating disorder in young women. Arch Gen Psychiatry.

[8] Rissmiller DJ, Rissmiller JH (2006). Evolution of the antipsychiatry movement into mental health consumerism. Psychiatr Serv.

[9] Bracken P, Thomas P, Timimi S (2012). Psychiatry beyond the current paradigm. Br J Psychiatry.

[10] Garrett CJ (1997). Recovery from anorexia nervosa: a sociological perspective. Int J Eat Disord.

[11] Serpell L, Treasure J, Teasdale J (1999). Anorexia nervosa: friend or foe?. Int J Eat Disord.

[12] Bjork T, Ahlstrom C (2008). The patient’s perception of having recovered from an eating disorder. Health Care Women Int.

[13] Sternheim L, Startup H, Saeidi S (2012). Understanding catastrophic worry in eating disorders: process and content characteristics. J Behav Ther Exp Psychiatry.

[14] Dawson L, Rhodes P, Touyz S (2014). “Doing the impossible”: the process of recovery from chronic anorexia nervosa. Qual Health Res.

[15] Moulding NT (2016). Gendered intersubjectivities in narratives of recovery from an eating disorder. Affilia J Women Soc Work.

[16] Lavis A (2016). A desire for anorexia: living through distress. Med Anthropol Theory.

[17] Hudelson PM (2004). Culture and quality: an anthropological perspective. International J Qual Health Care.

[18] Napier AD, Ancarno C, Butler B (2014). Culture and health. Lancet.

[19] Mattsson M, Topor A, Cullberg J (2008). Association between financial strain, social network and five-year recovery from first episode psychosis. Soc Psychiatry Psychiatr Epidemiol.

[20] Seebohm P, Gilchrist A (2008). Connect and include: an exploratory study of community development and mental health.

[21] Wand T (2015). Recovery is about a focus on resilience and wellness, not a fixation with risk and illness. Aust N Z J Psychiatry.

[22] Tew J (2013). Recovery capital: what enables a sustainable recovery from mental health difficulties?. Eur J Soc Work.

[23] Mol A (2009). Living with diabetes: care beyond choice and control. Lancet.

[24] Mol A (2008). The logic of care: health and the problem of patient choice.

[25] Musolino C, Warin M, Wade T & Gilchrist P. ‘Healthy anorexia’: the complexity of care in disordered eating. Social Sciences and Medicine. 2015;139:18–25. Doi:10.1016/j.socscimed.2015.06.030. 10.1016/j.socscimed.2015.06.030 26150064

[26] Lavis A, Abbots E-J, Lavis A, Attala L (2015). Careful starving: reflections on (not) eating, caring and anorexia. Careful eating: bodies, food and care.

[27] Butterfly Foundation. Investing in Need. Cost-effective interventions for eating disorders. Butterfly Foundation. 2015. Available at: http://thebutterflyfoundation.org.au/wp-content/uploads/2015/02/FULL-REPORT-Butterfly-Foundation-Investing-in-Need-cost-effectiveinterventions-for-eating-disorders-report.pdf (accessed 14 June 2016).

[28] Mond J, Hay P, Rodgers B (2004). Beliefs of women concerning the severity and prevalence of bulimia nervosa. Soc Psychol Psychiatr Epidemiol.

[29] Emerson RM, Fretz RI, Shaw LL (2011). Writing ethnographic fieldnotes.

[30] Corbin J, Strauss A (1990). Grounded theory research: procedures, canons, and evaluative criteria. Qual Sociol.

[31] Ezzy D (2002). Qualitative analysis: practice and innovation.

[32] Warin M. Abject Relations: Everyday Worlds of Anorexia. NJ: Rutgers University Press; 2010.

[33] Eli K. Embodying liminality: Eating disorders as a mode of coping with social suffering. Transcultural Psychiatry. July 2016. 10.1177/1363461518757799 29757091

[34] Hardin PK (2003). Social and cultural considerations in recovery from anorexia nervosa. A critical postructuralist analysis. Adv Nurs Sci.

[35] Rice C (2007). Becoming the fat girl: emergence of an unfit identity. Women’s Stud Int Forum.

[36] Berlant L, Metzl JM, Kirkland A (2010). Risky bigness: on obesity, eating, and the ambiguity of “health”. Against health: How health became the new morality.

[37] LeBesco K (2011). Neoliberalism, public health, and the moral perils of fatness. Crit Public Health.

[38] LaMarre A, Rice C (2015). Normal eating is counter-cultural: embodied experiences of eating disorder recovery. J Commun Appl Soc Psychol.

[39] Malson H, Bailey L, Clarke S (2011). Un/imaginable future selves: a discourse analysis of in-patients’ talk about recovery from an ‘eating disorder’. Eur Eat Disord Rev.

[40] Winace M, Mol A, Moser I, Pols J (2010). Care and disability: practices of experimenting, tinkering with, and arranging people and technical aids. Care in practice: on tinkering in clinics, homes, and farms.

[41] Buckett G, Volmer-Conna U (2015). Clinical practice guidelines for eating disorders – comments from the front line. Aust N Z J Psychiatry.

[42] Boughtwood D, Halse C (2010). Other than obedient: Girls’ constructions of doctors and treatment regimes for anorexia nervosa. J Commun Appl Soc Psychol.

[43] Touyz S, Le Grange D, Lacey H (2013). Treating severe and enduring anorexia nervosa: a randomized controlled trial. Psychol Med.

[44] Thaler L, Israel M, Antunes JM (2016). An examination of the role of autonomous versus controlled motivation in predicting inpatient treatment outcome for anorexia nervosa. Int J Eat Disord.

[45] Touyz SW, Beumont PJ, Glaun D (1984). A comparison of lenient and strict operant conditioning programmes in refeeding patients with anorexia nervosa. J Psychiatry.

[46] Hay PJ, Touyz S, Sud R (2012). Treatment for severe and enduring anorexia nervosa: a review. Aust N Z J Psychiatry.

